# Negotiating reform from within: workforce governance and the expatriate–Saudi interface in the SHSTP era

**DOI:** 10.3389/fpubh.2026.1902847

**Published:** 2026-09-18

**Authors:** Ahmed Alshehri, Asaad Abdulrahman Abduljawad

**Affiliations:** 1 Healthcare Management and Medical Informatics Department, College of Health Sciences, Umm Al-Qura University, Makkah, Saudi Arabia; 2 Department of Public Health, College of Health Sciences, Umm Al-Qura University, Makkah, Saudi Arabia

**Keywords:** expatriate healthcare workforce, health system reform, leadership, qualitative research, Saudi Health Sector Transformation Program (SHSTP), Saudization, vision 2030, workforce governance

## Abstract

**Background:**

Saudi Arabia's Health Sector Transformation Program (SHSTP), introduced under Vision 2030, aims to improve health system performance while increasing the participation of Saudi nationals in the healthcare workforce. Although expatriate healthcare professionals continue to play a central role in service delivery, little is known about how workforce reform is experienced and governed within mixed Saudi–expatriate healthcare settings during this period of transformation.

**Methods:**

This paper presents a secondary analysis of data collected as part of a broader doctoral study examining implementation of the SHSTP. The analysis draws on 53 semi-structured interviews with physicians (*n* = 30) and patients (*n* = 23), alongside a review of relevant policy and strategic documents. Data were analyzed using reflexive thematic analysis informed by an adapted Health Policy Analysis Triangle, with particular attention to actors, implementation processes, technology, and organizational culture.

**Results:**

Five interrelated themes were identified. First, collaborative professional cultures supported mutual learning and adaptation during reform. Second, authority was often negotiated between formal managerial structures and expertise-based influence. Third, leadership roles were reshaped by new accountability expectations and digital performance systems. Fourth, participants described ongoing capacity and competency challenges, highlighting the importance of mentoring and professional development. Finally, concerns about retention and motivation were linked to appraisal practices, contractual uncertainty, and professional identity. Across all themes, reform emerged as an ongoing organizational process shaped by everyday professional interactions rather than the straightforward implementation of policy directives.

**Conclusion:**

Workforce reform under the SHSTP is shaped by everyday professional relationships, leadership practices, and organizational dynamics. Strengthening mentorship, leadership development, transparent appraisal processes, and knowledge-retention mechanisms may support workforce stability while advancing localization goals. These findings contribute to a deeper understanding of workforce governance in health systems undergoing large-scale reform.

## 1 Introduction

Health system reforms depend fundamentally on the workforce responsible for implementing them. Healthcare professionals translate policy goals into everyday practice, shaping care coordination, service continuity, and accountability across healthcare organizations. Consequently, the success of large-scale reform initiatives depends not only on policy design but also on how the workforce is governed, supported, and mobilized during implementation (1).

This is especially true in Saudi Arabia with the Saudi Health Sector Transformation Program (SHSTP) which was established in 2016 under Vision 2030. The SHSTP aims to re-engineer healthcare service delivery via cluster-based approaches, enhance primary care, build up digital health technologies and enhance efficiency and quality within the system. While the outcomes of these reforms are typically measured in terms of patient outcomes or system performance, the ability of the health workforce to adjust to new structures, leadership and practice expectations is a key determinant of the success of the reforms. Thus, understanding the role of manpower in SHSTP is crucial in understanding the process of reform in practice (Figure 1).

**Figure 1:**
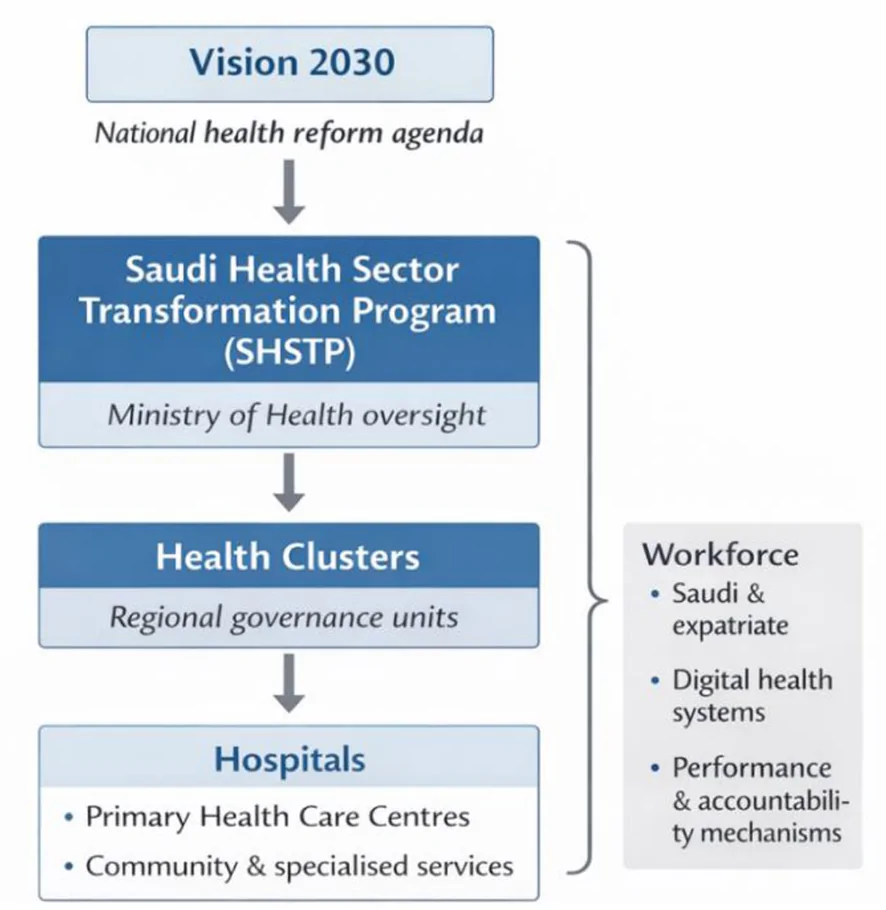
Organizational structure of the Saudi Health Sector Transformation Program (SHSTP).

The figure depicts the multi-layered organizational structure of the SHSTP, showing how national health reform under Vision 2030 is translated through health-sector structures and health clusters. These organizational configurations shape the implementation and governance of workforce reforms.

Workforce localization is a fundamental part of Vision 2030, which is often expressed in Saudization policies, which seek to boost the national workforce's representation in various sectors, such as healthcare. The health system policies focus on building the capabilities of Saudi clinical and managerial workforce, succession planning, and sustainability of the workforce. The localization is seen as a strategic approach in the face of demographic change and dependency on the labor market, and as a way of enhancing institutional resilience and continuity of care. The national workforce is a key element of sustainable health system reform, in every policy document (2).

In the SHSTP, the Saudization targets are related to the broader reform aspirations of better governance, accountability and integration of services. Health clusters should facilitate leadership development, training and career growth of Saudi professionals and also provide an opportunity to integrate new care delivery models. Localization is thus not seen as an employment policy in its own right, but as part of the transformation of the health sector. But, bringing these aspirations to fruition in terms of how they are put into practice with the existing workforce and service delivery needs is a different matter.

Meanwhile, Saudi Arabia's health care system still heavily depends on foreign health care workers. Expatriate physicians, nurses, and allied health personnel continue to play a vital role in delivering services, especially in clinical specialty roles and senior positions. It is recognized in Ministry of Health statements, as well as the need to transfer knowledge, mentor and build skills in the national workforce. Expatriate professionals are then placed in a situation where they are both service providers and capacity builders in the restructured health organizations, under the SHSTP. This puts ongoing pressure on the need for localization goals and the ongoing requirement for expatriate skills, especially in newly formed health clusters (3, 4).

These dynamics create governance problems which affect how reform is experienced within organizations. Localization and reliance on expatriates exist side by side in the same institutional contexts and impact on authority, decision making and professional identity. This tension is not an inconsistency in policy, but a process of negotiation in which actors react to the pressures for reform, the constraints of operation and the realities of clinical practice in a pragmatic manner. The nature of the negotiations will have significant consequences for the stability of the workforce, effectiveness of leadership and the long-term viability of reform.

While workforce issues are prominent in policy discussions, there are few analyses of the SHSTP and similar reforms conducted from a workforce perspective. Workforce is typically considered a contextual issue rather than an active area of governance. Fewer studies have focused on the experience of healthcare professionals in the process of reform, the negotiation of authority in hybrid workforces, and the interpretation and adaptation of new expectations in times of continual reform (5).

The aim of this paper is to fill this gap by examining the governance of healthcare workforce in the context of SHSTP, as experienced by Saudi and expatriate healthcare workers. The study is based on a qualitative analysis of policy documents and clinician and manager interviews and explores the negotiation of the reform of the workforce in practice. The analysis is concerned with the everyday organizational processes, leadership and professional relationships that influence the implementation of reform in addition to the formal design of reform. By doing so, the paper aims to help develop a more complex understanding of how health system transformation is achieved through workforce governance during times of significant health system transformation.

## 2 Analytical and theoretical lens

This study takes an analytical approach that focuses on the actors and process during policy implementation, to explore the implementation of the Saudi Health Sector Transformation Program (SHSTP) in terms of workforce reform. Health system reforms do not necessarily happen in a linear manner, as a translation of policy design, but are negotiated, interpreted and enacted in the day-to-day work of those who are charged with implementing change. A lens for analysis of these dynamics is therefore crucial to understanding the operation of the workforce governance during large scale transformation.

The analysis is informed by the Health Policy Analysis Triangle developed by Walt and Gilson (6), which views policy as the product of content, context, process and actors. The framework has been successfully used in health policy research, but the actors and process aspects are highlighted in this study. This focus is due to the study's interest in the interactions between healthcare professionals, managers and institutional leaders with the reform process, and in how policy intentions are realized in practice in the organization in restructured healthcare systems. The framework is not a policy *per se*, but rather a process of reform, allowing for consideration of the process as negotiated and dynamic, with multiple actors involved.

This study builds on an adapted version of the policy triangle created in previous research and adds the factors of technology and culture as cross-cutting influences. In the context of the SHSTP, digital health technologies are not just tools, but agents of change in the way that health care is organized, who is responsible for what, and how services are delivered. Likewise, institutional and professional cultures shape the understanding, opposition and adaptation of reform in the day-to-day work. By incorporating technology and culture into the analytical lens, one can focus more on how actors interact with the processes of reform while simultaneously not ignoring the influence of technology and culture on the governance.

In this adapted context, actors are defined as Saudi and expatriate clinicians, middle managers and senior leaders within health clusters. The actors have different roles of power and influence and their reaction to reform is influenced by their professional background, contractual status and organizational role. Importantly, the analysis does not assume uniformity of interest and motivation within the groups. Instead, it explores the ways in which actors engage with the demands for reform in a pragmatic way, taking into account the policy expectations, clinical duties, organizational pressures and professional identities.

The process dimension emphasizes the long-term implementation of reform, both formally and informally. This encompasses new governance structures, leadership and expectations under the SHSTP and the day-to-day practices in which this is implemented. The analysis focuses on processes to reflect how the workforce localization, leadership development and capacity building processes are implemented in health clusters, and how they are experienced by those who provide care.

The analysis selectively borrows ideas from health human resource and organizational governance literature to further shed light on the dynamics of the workforce governance. Distributed leadership is employed to describe how authority and responsibility are distributed and not just in formal leadership positions (7). The concept of leadership is very relevant to the examination of how leadership is performed in restructured health systems, in which the leadership is often expressed through teams, networks and professional relationships, and how responsibilities are shared and negotiated across organizational levels.

The analysis is also based on the concept of hybrid governance. Hybrid governance is the presence of several forms of governance, including hierarchical, professional and performance-based (8). Within this hybrid, the state's supervision, management practices, and professional standards converge, health clusters work under the SHSTP. These configurations influence the implementation and resistance to workforce governance in an organization, especially when the workforce is composed of people from different nationalities.

Last, the idea of institutional isomorphism offers an explanation for the ways that organizations react to pressures for reform by mimicking one another in their structures, practices or discourses in response to regulatory and normative expectations (9). While implementation of the reform varies across the SHSTP, health clusters are encouraged to link to nationally defined reform models and performance frameworks, which can impact on workforce governance practices. This idea can be used to describe convergence trends within organizations and to leave room for the local variations of practice.

Combined, these perspectives enable an analysis of the process of workforce governance as an emergent and negotiated process, not a direct result of policy design. The overall framework for the analysis of actors and process is the adapted policy triangle, which is complemented by concepts of HRH and governance for the analysis of how authority, leadership and professional relationships are established in the context of reform.

The analytical framework combines the adapted Health Policy Analysis Triangle with health workforce and governance concepts to examine workforce governance under the SHSTP. It emphasizes actors and implementation processes, while treating technology and organizational culture as cross-cutting influences on workforce governance. Distributed leadership, hybrid governance, and institutional isomorphism are incorporated as complementary lenses for examining how authority, professional relationships, and organizational practices shape the ongoing negotiation of workforce reform.

## 3 Methods

### 3.1 Study design and data sources

The data for this study were qualitative and were produced on health system reform in the Saudi Health Sector Transformation Program (SHSTP). Qualitative policy and organizational analysis (POA) was used to gain insight into the actual process of workforce governance and reform, focusing on the experiences, interactions and interpretations of actors in the process of implementing it. This is appropriate for the study of complex reforms where outcomes are not only dependent on the policy design, but also on professional behavior and organizational context and governance processes (10). The present study represents a focused secondary qualitative analysis of data generated within a broader doctoral research project examining implementation of the Saudi Health Sector Transformation Program (SHSTP). While the parent study employed a mixed-methods design to investigate multiple dimensions of SHSTP implementation, including patient-centered care and chronic disease management, the current analysis draws specifically on qualitative interview data and policy documents to explore workforce governance and the Saudi–expatriate interface during health sector reform.

The analysis was based on two main types of qualitative data sources:

1. **Semi-structured interviews** with the physicians and patients in the health care settings that are undergoing restructuring as part of the SHSTP.
2. **Policy and strategic documents** such as Vision 2030 health sector documents, Ministry of Health workforce policies and guidance for the implementation of SHSTP that are relevant to the governance of the workforce and service delivery.

Interviews complemented policy documents to analyze reform as a formal policy process as well as an organizational process experienced by the people (11).

### 3.2 Interview sample and sampling strategy

The interview data consisted of 53 semi-structured interviews: 30 physicians and 23 patients. Physicians were from a variety of professional seniority and clinical roles in restructured health care environments and patient participants were from services impacted by SHSTP related organizational change.

The physicians were purposively sampled to ensure variation in their professional role, their organizational responsibility and nationality. Both Saudi and expatriate physicians were included, as the Saudi health workforce is a hybrid of both national and expatriate doctors and localization dynamics are central to this study. The sampling focused on information richness and analytic relevance, which is in line with the principles of qualitative health policy research, rather than aiming for statistically representative sample sizes (12).

To gain insight into the experiences of workforce governance and organizational change at the point of care, patient interviews were included. Although the primary focus of this paper was workforce characteristics, the patient voice is important to consider in understanding the impact of workforce characteristics, continuity of care, and professional interactions on the patient's experience of the reform process.

Eligible patient participants were identified through hospital gatekeepers using hospital records and predefined study eligibility criteria. The patient information sheet, consent documentation, and eligibility criteria were provided to the gatekeepers, who identified potentially eligible patients in the participating hospitals. Interview arrangements were subsequently made with eligible patients who agreed to participate, and written informed consent was obtained before the interviews.

Patient eligibility required participants to be adults aged 18 years or older, diagnosed with at least one complex chronic condition, and receiving care in the selected hospitals for at least 6 months. Participants were also required to provide informed consent. Patients with acute or terminal illnesses unrelated to chronic conditions were excluded, as were individuals unable to provide informed consent because of cognitive impairment or language barriers.

Variation in patient experiences was considered during sampling by including participants from both clustered and non-clustered hospital settings and across different clinical services and chronic conditions. The sample encompassed patients receiving care for conditions including cancer, renal failure, cardiac disease, diabetes, hypertension, and cardiovascular disease. Variation in age, gender, socioeconomic background, and duration of care was also considered to capture differences in patients' experiences of organizational and service changes associated with the SHSTP. Physicians with varied clinical and organizational responsibilities, including leadership and management roles, from SHSTP-affected settings (includes Saudi and expatriate clinicians) (Table 1).

**Table 1:** Overview of the participants in the interviews.

Participant group	Description	*n*
Physicians	Physicians in varied clinical, managerial and leadership roles from SHSTP-affected settings (includes Saudi and expatriate clinicians)	30
Patients	Patients interviewed from SHSTP-affected settings	23
Total interviews		53

### 3.3 Policy document selection

The policy documents were chosen because they are relevant to the issues of workforce governance, localization and organizational reform under the SHSTP. These comprised Vision 2030 health sector strategies, policy statements of the Ministry of Health, and documents of the SHSTP program on workforce development, leadership and restructuring of services. Documents were examined to see if there were any dominant policy narratives on workforce reform and to situate the findings of the interviews in the formal reform agenda.

### 3.4 Data collection

Based on the analytical framework and literature from the study, semi-structured interview guides were created (13). Interview questions focused on the participants' experiences of organizational change, on their perceptions of professional roles and authority, leadership arrangements and on their experiences of Saudi and expatriate staff working together. Patient interviews were directed toward continuity of care, communication and service delivery changes as a result of workforce and organizational restructuring.

All interviews were taped, with the permission of the interviewees, and transcribed exactly as they were recorded. The flexible interview format enabled participants to bring up issues they deemed important and also ensured that themes that were important for the workforce governance and reform processes were covered.

### 3.5 Data analysis

Thematic analysis was used to analyze the interview transcripts and policy documents and was an iterative and reflexive process (14). There were various steps in the analysis. The first step was to repeatedly read the transcripts to familiarize oneself with the data. This was followed by initial coding to identify parts of text that are related to workforce governance, leadership, authority, professional identity and implementation of reform. Themes were identified within the participant accounts and themes from the inductive analysis were then used to guide the deductive analysis of the actors and process dimensions of the policy analysis framework.

Codes were then reviewed, streamlined and categorized into larger groups to allow for patterns to be seen between physician and patient accounts. In this way, themes were elaborated that highlighted significant issues in the governance of the workforce in the context of the SHSTP, such as professional relationships, negotiation of authority, leadership in the face of transformation, workforce capacity and competency building and workforce retention issues. In parallel, policy documents were analyzed to look at alignment and divergence with lived organizational experiences as part of the formal reform narratives.

Patient accounts were used primarily to examine the service-level consequences of workforce governance rather than to infer internal workforce dynamics directly. Their accounts provided an external perspective on how professional collaboration, authority arrangements, leadership practices, workforce capacity, and staff stability were experienced through continuity of care, communication, coordination, responsiveness, and changes in providers. These accounts were interpreted alongside physician accounts to identify areas of convergence and divergence, thereby showing how workforce governance within mixed Saudi–expatriate settings could translate into experiences at the point of care.

### 3.6 Rigor and analytic validation

To increase the rigor of the analysis several strategies were used. The coding and interpretation process was facilitated by peer debriefing and themes were discussed and developed with a research peer with experience in qualitative health policy research. This process facilitated reflexive engagement with the data and challenged assumptions in interpretation (15). Triangulation of the interview data with policy documents also contributed to the credibility of the findings, by placing the participants' accounts in the context of the policy.

### 3.7 Reflexivity

Reflexivity was a part of the process of research. The researcher was in a partial insider/outsider position to the study context, as both an insider and an outsider, having a familiarity with the Saudi health reform context, and being an outsider as a researcher. This positioning influenced who was included and excluded in the study and how results were interpreted, and required constant reflexivity regarding how previous knowledge, professional experience and assumptions could have impacted on analytic choices (16).

### 3.8 Ethical considerations

Ethical approval for the parent doctoral study was obtained from the Research Ethics Committee of the College of Health Sciences at Swansea University (Protocol 280818) and from Umm Al-Qura University, Saudi Arabia (Protocol 2019/099). Written informed consent was obtained from all participants prior to data collection. All interview data were anonymised prior to analysis, and participant confidentiality was maintained throughout the study. The present paper is based on a secondary analysis of data collected as part of the broader doctoral research project.

## 4 Findings

The themes identified in the analysis of the interviews were interrelated and revealed five themes that shed light on the experience and negotiation of workforce governance and reform under the Saudi Health Sector Transformation Program (SHSTP). These themes reflect the daily life, conflicts and adaptations that bring about reform in mixed national and expatriate workforces.

The five core themes that arose from the analysis are shown in the figure and are interrelated. The network emphasizes the workforce governance process as an interconnected and dynamic process, influenced by inter-relationships between organizational culture, authority, leadership practices, capacity development and workforce stability throughout the health system reform journey.

### 4.1 Collaborative professional cultures: initiative diversity and mutual learning

The physician and patient interviews both indicated that the reform environment was one in which there was greater collaboration between professionals and organizations. Reform initiatives in clustered settings seemed to have led to better structured interactions across departments, disciplines and facilities, especially when new care pathways and digital systems needed to be coordinated outside of the traditional silos. Doctors reported that working together became part of their daily work, sometimes as a result of team goals and/or system-wide procedures.

In mixed teams (Saudi and expatriate), the process of working together was often described as a process of learning from each other. Expatriate doctors spoke about bringing clinical experience, procedural knowledge and experience of international best practice, whereas Saudi doctors spoke of their increasing involvement in the context of care delivery in the country's priorities and patient expectations. Knowledge transfer was often seen as a process of mutual learning, rather than a unidirectional flow of information and knowledge, and was influenced by the day-to-day problem solving and adapting to new organizational requirements.

“We have experience from other systems, but the Saudi colleagues help us to understand how things really work here, you learn from each other every day.” (Expatriate physician, clustered hospital)

“Clinical guidelines are important, but we need to consider our patients' expectations and the system we are working in.” (Saudi physician, clustered hospital)

This impression was confirmed by patients' experiences, especially with regard to continuity of care. Some patients reported better communication and referral processes between services, better coordination of clinicians and clearer communication between clinicians. These experiences were often not attributable to individual providers, but were associated with teamwork, indicating that team cultures were becoming more apparent at the point of care.

“Now the doctors are able to talk to each other, I don't have to repeat my case each time.” (Patient, clustered hospital)

However, collaboration was not universal. Collaborative practices were found to be more advanced in clustered hospitals than in non-clustered hospitals, where there was a continued lack of organization. Others emphasized the importance of local leadership and interpersonal relations as a key factor in achieving collaboration, rather than its institutionalization. Despite these differences, a common professional culture of collaboration was found to be a key characteristic of the implementation of reform, facilitating adaptation and learning in the face of change in the organization.

### 4.2 Negotiating authority: line management conflicts between nationals and expats

A second theme focused on the negotiation of authority in mixed national/expatriate workforces. Participants talked of a more nuanced picture than policy discourses suggest focusing on localization and Saudi leadership development, but the formal authority, professional expertise and organizational influence did not always converge. In some reports, Saudi physicians occupied formal positions of management or supervision, and expatriate physicians had a high level of informal authority in the clinic, as a result of their clinical experience or length of time working in the clinic.

The lack of a match between formal and experiential authority was often expressed by participants:

“On paper, it is a Saudi manager, but in reality people still go to the senior expatriate doctor for decisions because of experience.” (Expatriate physician, clustered hospital)

It was sometimes unclear to expatriate participants what their reporting relationship was, especially when it was with less experienced national colleagues. On the other hand, Saudi participants mentioned the difficulty of asserting leadership in situations where expatriate employees had more autonomy and/or informal power:

“Others have been here much longer and are accustomed to making their own decisions, it is not easy to be the leader.” (Saudi physician, managerial role)

These conflicts were not always settled in formal settings but were sometimes negotiated and accommodated. Participants talked about strategies that they used to ensure effective relationships: Shared decision-making, informal consultation, and selective delegation:

“We attempt to talk about it together and agree, otherwise there are issues in the team.” (Saudi physician, clustered hospital)

These practices served to maintain service delivery but also demonstrated the fragility of authority arrangements in the process of reform, especially when there is no clarity about the roles and no leadership support.

Importantly, the patients were not generally aware of these internal negotiations, but they were an indirect reflection of the consequences. Delays or lack of communication were reported by patients where authority was in conflict, and team working was not functioning. The collaborative management of authority was associated with patients' perception of smoother care processes. This theme highlights the importance of tensions within the workforce governance process, which can have a significant impact on the service experience, although these tensions are likely to be internal.

### 4.3 Leadership under transformation: shifting accountability and digital bureaucracy

Leadership was a key issue that arose in interviews, especially with regard to the new accountability for the SHSTP. The participants spoke of a more complex and challenging leadership role due to the adoption of cluster governance, performance monitoring and digital reporting systems. Managers felt they had greater accountability to central authorities and were expected to help staff with the constant change in the organization.

The digital systems had a great impact on transforming the leadership practices. Reports of the use of electronic dashboards, performance indicators, and reporting requirements were common themes that had a positive impact on oversight, but also added administrative burden:

“Nowadays, most of our time is dedicated to reporting and updating systems, instead of being with the team; leadership is more about meeting indicators than being with the team.” (Manager, clustered hospital)

Some leaders reported that they felt “bureaucratic” in the digital world and had less time for clinical level problem-solving, mentoring and relational leadership.

There was a wide range of experiences among physicians about being in a leadership position. Some leaders were seen as facilitators to assist teams to meet demands of reform and adjust processes. In others, leadership was felt to be distant and/or compliance focused, with the emphasis on achieving targets, not supporting professional practice:

“With a supportive leadership, it becomes easier to change but sometimes it's just about targets and not people.” (Physician, clustered hospital)

These differences were frequently explained as being due to the different leadership styles, not to role definitions.

Patients' experiences reflected the downstream impact of leadership practices, in terms of service responsiveness and communication. Patients felt more confident in the delivery of care when teams were stable and processes were clear, where the leadership supported them. Patients reported inconsistencies in the quality of services when there was a lack of leadership. Leadership in transformation thus became one of the important ways in which reform priorities became a reality on a day-to-day basis.

### 4.4 Capacity and competency gaps: training, mentoring, and local empowerment

Capacity development was a key theme and was closely related to Saudization goals. Significant strides were made in clinical training and professional development opportunities for Saudi clinicians, especially in clustered settings, which were widely recognized by the participants. Structured training programs, mentoring relationships and leadership development programs were mentioned as becoming more prominent elements of reform implementation.

Often expatriate physicians would be placed as mentors to help build the skills and confidence of Saudi physicians. This was seen as a positive role by many, but some were concerned about the viability of informal mentoring if it was not recognized or afforded protected time:

“We are happy to mentor but sometimes it is not recognized or supported, it's on top of our workload.”(Expatriate physician, clustered hospital)

Saudi participants also emphasized the importance of mentoring, however, there were also differences in access and quality in various settings:

“Some departments are very supportive but in others it is expected that you take responsibility very quickly without adequate preparation.” (Saudi physician)

Participants still noted that there were areas of competency that they felt needed to be improved, especially in the specialized clinical areas and in managerial positions. The gaps were sometimes described as ‘transitional challenges' not failures, but rather a result of the pace and scale of reform. But there was a concern about the potential of overloading emerging Saudi leaders with the responsibility without support, which could lead to loss of confidence and retention:

“You get promoted quickly but then sometimes when you‘re promoted, you're not ready for the job.” (Saudi physician, early leadership role)

Patients' views highlighted the need for capacity building, specifically in terms of consistency of care. With successful training and mentoring, patients reported that providers were confident and that they remained the same throughout their visits. Where gaps existed, patients commented on the need for support from certain people and not systems. Capacity and competency development became therefore the key in workforce empowerment and in ensuring service stability.

### 4.5 Retention and motivation dilemmas: contracts, appraisal fairness and identity balance

The last theme was on retaining and motivating a workforce undergoing reform. Participants expressed concerns about contractual issues, appraisal and career progression in the context of the SHSTP. Expatriate physicians voiced concerns regarding role security, inequities in appraisal processes and contract renewal, which at times had an impact on their motivation and long-term commitment:

“You don't know if your contract is going to be a good one, or if the rules are going to change, it's never really certain, that has an impact on people's commitment.” (Expatriate physician)

Saudi participants tended to be more positive about their job prospects but also mentioned pressure to meet the expectations of the reforms in a timely fashion. A major issue was the need to balance the demands of professional identity, leadership aspirations and service needs, especially for the transitioning professionals. Some participants commented that it was important to ensure that there was adequate preparation for rapid promotion to avoid loss of confidence and job satisfaction.

“There are a lot of opportunities, but there's a lot of pressure, too. You‘re expected to lead even if you're still learning.” (Saudi physician, early leadership role)

Perceptions of fairness and recognition were highly related to motivation. From all groups, there was a consensus that it was important to have transparent appraisal systems, clear criteria for promotion and recognition of achievements. When these elements were felt to be missing, the participants said that they were disengaged or would look for other opportunities.

These dynamics were indirectly expressed by patients' reports of continuity and turnover of staff. Turnover or multiple changes of provider was a negative experience and impacted trust and satisfaction. Workforce issues and retention and motivation issues are not just workforce issues but are also important aspects of sustainability of the reforms.

These five themes together provide a picture of workforce governance as a process that emerges and is negotiated, resulting from collaboration, authority, leadership, capacity building and motivation. The results show that reform is implemented in the day-to-day organizational practices, not just in the design of policies, and demonstrate the need to pay attention to workforce experiences to better understand health system reform.

## 5 Discussion

This study used a conceptually driven qualitative approach that focused on the actors and process as key mechanisms of implementing reform under the Saudi Health Sector Transformation Program (SHSTP). The results show that workforce reform under the SHSTP is not achieved through the design of the policy, but rather through continuous organizational negotiation that entails professional collaboration, power dynamics, leadership, capacity building, and motivating the workforce. These results are analyzed from the organizational and policy implementation perspectives, to better understand the implementation of health system reform in practice in the context of hybrid workforces and the changing institutional landscape.

### 5.1 Workforce governance as an organizational process not a policy outcome

Across all five themes, workforce governance emerged as an ongoing organizational process shaped through everyday interactions rather than as a direct outcome of policy. This finding aligns with health policy research that challenges linear models of reform implementation, particularly in low- and middle-income and transitioning health systems where policies are continually interpreted, negotiated, and adapted in practice (17, 18). In this context, policy frameworks provide direction, but organizational processes ultimately shape how reform is enacted.

Collaborative professional cultures are strongly evident and suggest that reform efforts can create new cultures of team working, particularly when there are structural changes that make services more interdependent. Collaboration also is a practical solution to system restructuring, resource limitations, and system performance pressures, as has been seen in LMIC health reforms (19). But as the results in this research demonstrate, collaboration is not necessarily part of the governance structure and depends on the leadership capacity and organizational culture.

### 5.2 Negotiating authority within hybrid workforces

One of the key aspects of the negotiation of authority between Saudi and expatriate professionals that is not often explored when evaluating reform is the issue of the negotiation of authority. Saudization is envisioned as a strategy toward sustainability and empowerment of the nation, but it also collides with existing professional hierarchies and expertise-based authority. The findings in this study indicate that managerial positions are not necessarily effective in providing governance, especially when clinical expertise and tacit knowledge is held by expatriate personnel.

Similar patterns have been observed in other LMIC and Gulf health systems where there are both policies of localization/nationalization and ongoing use of expatriate expertise (20). In this context, authority is often negotiated in *ad hoc* ways, often by means of interpersonal relationships and pragmatic accommodation. Such arrangements can help to maintain service delivery but can also create confusion about accountability if they are not accompanied by a clear definition of roles and development of leadership (21).

### 5.3 Leadership under transformation and digital governance

Leadership was identified as a key way in which priorities for reform was “delivered” in the organization. Participants noted that the roles of leaders are becoming increasingly influenced by the digital governance tools, performance monitoring and reporting demands of the SHSTP. This is part of a broader trend in health systems around the world to become more digital, shifting accountability to information systems and performance measures (22).

The findings indicate that digital tools can be used as a tool to provide oversight and transparency but can also limit relational leadership if not supported by good management practices. Coordinating beyond the hierarchical roles of leader to leader is essential in cluster-based structures, as leaders must be able to do so across services, professions and organizational boundaries. The participants indicated higher levels of collaboration and stability in the workforce, and adaptive, distributed leadership during change, as well, when leadership capacity was high, consistent with other reforming health systems that have suggested that adaptive and distributed leadership is crucial during change (23).

### 5.4 Capacity development as a transition governance challenge

Capacity and competency building was a key achievement and challenge in the SHSTP. Training, mentoring and leadership development of Saudi professionals are in line with localization goals and best practices in workforce reform internationally (24). The results indicate that these types of programs work best when they are part of a formal mentoring program, rather than just informal mentoring.

As with other LMIC reforms, however, there are risks during the transition period when the expansion of roles is outstripped by capacity development and support (25). Expatriate mentoring, if overused, without recognition and workload adjustment, can be a strain on the mentor and emerging leader. The results highlight the need to design capacity building programs in line with governance structures that facilitate a smooth transition of roles and confidence building. For such programmes to be sustainable, mentoring should be integrated into routine organizational practice rather than treated as an additional informal responsibility. This could include protected time within clinicians' workloads and explicit recognition of mentoring contributions within performance appraisal and promotion processes.

### 5.5 Retention and motivation as measures of the sustainability of reform

The retention and motivation issues are important lenses to evaluate the sustainability of workforce reform. The results show that uncertainty of contracts, appraisal and career progression has an impact on both the expatriate and the national staff, albeit in different ways. The issue of inequities and insecurity in the health systems has been well reported in the context of reforming health systems, as it can affect the commitment and continuity of the health system's workforce (26).

Importantly, the relationship between workforce stability and service experience was indirectly reinforced in this study, with links between workforce turnover and disengagement, and between continuity of care. Retention and motivation are thus not only outcomes of the workforce, but indicators of governance and as such have implications for the effectiveness of the reform and patient trust.

### 5.6 Analyzing the results in the adapted policy triangle

The results of this study confirm that actors and process are key in determining the outcomes of reform as seen through the adapted Health Policy Analysis Triangle developed in Publication 1 (6, 7), Policy content and context set the boundaries of reform, but it is actors' interpretations, negotiations, and organizational practices that make reform happen. The use of technology and organizational culture as cross-cutting factors are important in understanding the differences in experiences that can be found across similar reform structures.

The five themes are conceptually linked together in Figure 2 and are conceptually linked to each other in Figure 3. They both reinforce the idea of a dynamic, relational and iterative view of workforce governance, as opposed to a linear and fully designed view of workforce governance.

**Figure 2:**
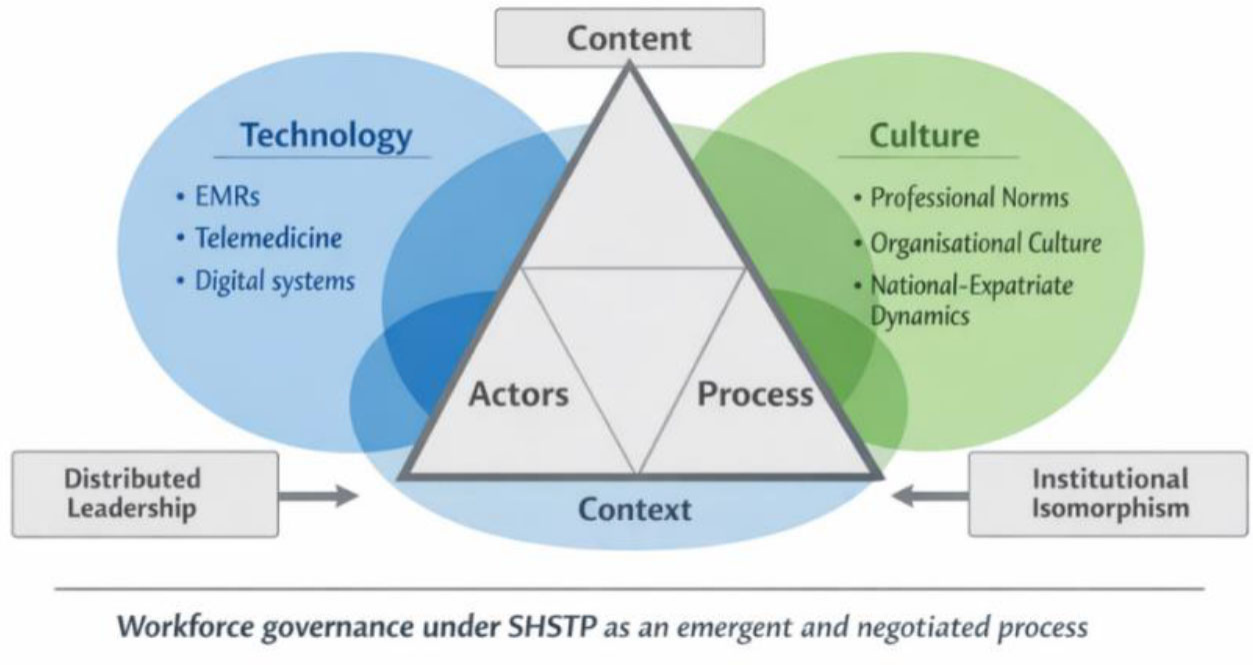
Analytical framework for analyzing the workforce governance in the Saudi Health Sector Transformation Program (SHSTP).

**Figure 3:**
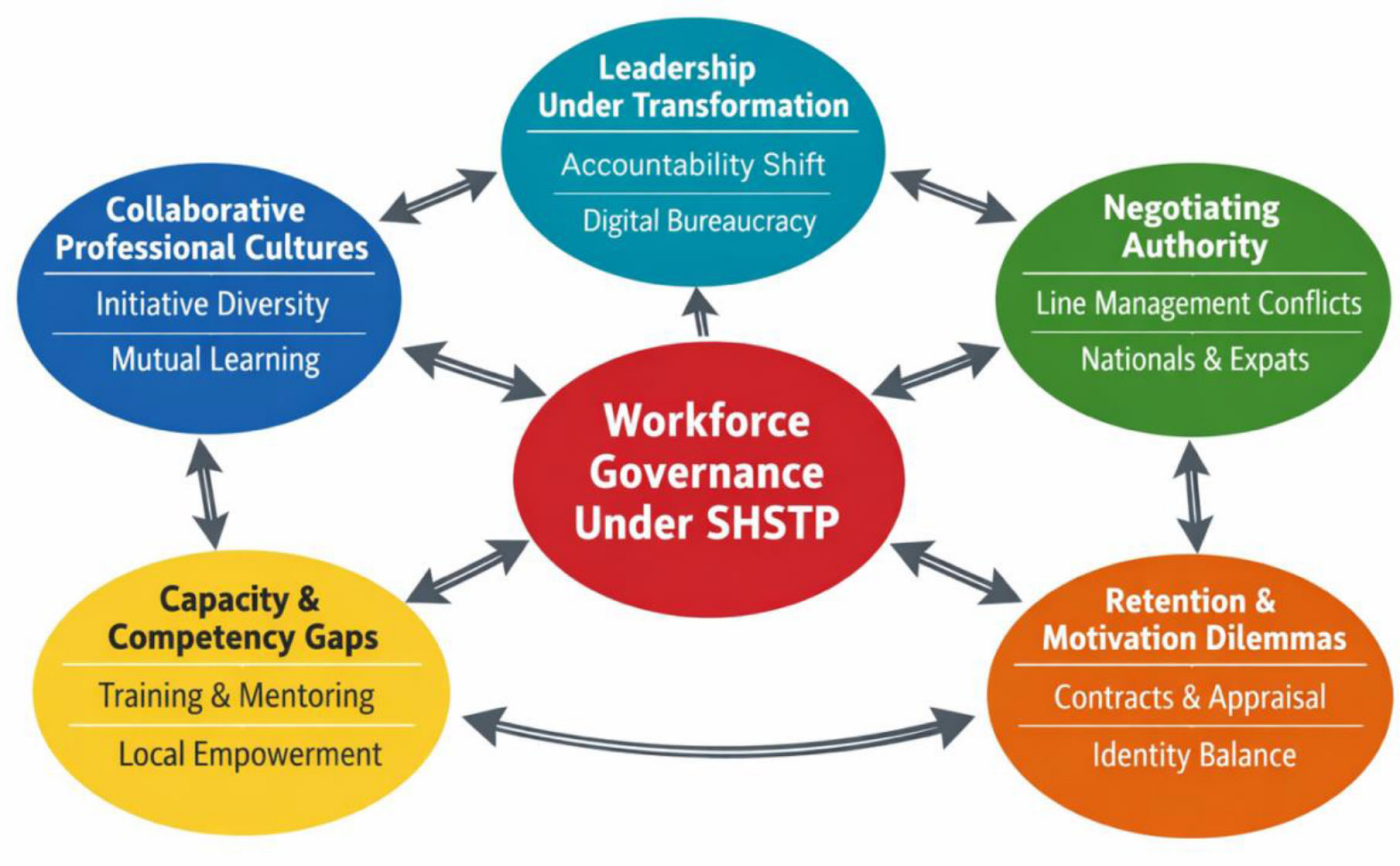
The Saudi Health Sector Transformation Program (SHSTP)'s thematic network of workforce governance. The network illustrates the five interrelated themes identified in the analysis and their relationship to workforce governance under the SHSTP. Arrows indicate conceptual interrelationships and reciprocal influence among the themes rather than causal relationships.

### 5.7 Implications for SHSTP and comparable reforms

The results indicate that workforce governance needs to be a constant focus for the SHSTP and not limited to the formal localization goals and structural reorganization. Stable governance in the reform process is essential to having clear authority structures, leadership development, transparent appraisal systems and formalized mentoring systems. Meanwhile, the reform approaches should also not neglect the importance of expatriate expertise in the course of transition.

This study also helps to inform discussions about workforce governance in reforming health systems, not only in Saudi Arabia, but also in other settings around the world, such as LMIC and hybrid settings. It highlights the role of organizations and the interactions between actors, providing insights that are relevant to countries that are working toward localization, decentralization and/or digitalization within the context of a health system reform agenda.

## 6 Policy implications

This study has several policy implications for the Saudi Health Sector Transformation Program (SHSTP) that can be taken. The analysis does not call for a redesign of the reform architecture but rather identifies specific governance interventions that can enhance the implementation of the reform, by focusing on how actors and processes work within the existing architecture. This is especially true when considering the need to localize goals in the context of hybrid workforces and ongoing need for expatriate expertise.

### 6.1 Strengthening mentorship through structured Saudi–expatriate pairing

The results of the analysis were consistent in that the informal knowledge transfer between expatriate and Saudi professionals was found to be very significant. These mentoring relationships are important for capacity development, but are often informal and are not sustainable, and can be an unequal burden on individuals. Therefore, the efforts of the policy should be directed toward formalizing the structured mentorship pairing between Saudi and expatriate clinicians and managers.

These programs should be more than just a one-off initiative and be integrated into the cluster and hospital workforce governance. To ensure mentorship is a meaningful and effective way to develop skills and readiness for leadership, it is important to have clear expectations, defined time allocations, and recognition in appraisal systems. Importantly, formal mentorship should be viewed as a tool to help with the transition of roles, not as a replacement for localization, but as a tool to help make the transition to the new role gradually and effectively while keeping in mind the institutional knowledge.

### 6.2 Creating a leadership development continuum in an integrated way

The complexity of leadership, under the SHSTP, has grown more complicated due to cluster governance, digital accountability systems and demands for cross-organizational coordination. The results indicate that, in addition to being formally appointed as a leader, leadership effectiveness is related to preparation, support, and development. One of the important policy implications is that there must be a clear leadership development pathway that is consistent with the policy goals for reform.

This pipeline should include early identification of potential leaders, a series of development opportunities and combine clinical, managerial and digital skills. Policy frameworks should focus on readiness, mentoring and experiential learning, rather than promoting fast-tracking of people into positions of leadership based on localization targets. Linking leadership development to cluster level governance would also facilitate consistency of leadership development across settings and provide for local adaptation.

### 6.3 Implementing equitable appraisal and career progression frameworks

Fairness in appraisal and career progression became a key issue in the motivation and retention of the workforce. Expatriate and Saudi professionals were concerned about these issues, but in different ways. Policy responses should thus focus on building clear and fair evaluation systems and making sure that there is a relationship between expectations of performance, professional development and promotion.

The use of common evaluation criteria within clusters and flexibility to consider the specific context of the roles could help minimize the sense of arbitrariness and inequity. Stable workforce would be further supported by clear communication of career pathways, especially when there is a change in the organization. It is important to note that appraisal systems should reward efforts to support mentoring, leadership development and organizational learning, as well as clinical output or compliance measures.

### 6.4 Aligning digital skills training with reform goals

SHSTP has introduced digital systems that have been pivotal in driving accountability, reporting and performance management. The results also indicate that digitalization can also result in additional administrative burden and lead to a greater gap between leadership and frontline practice if not supported by adequate capacity building. Policy interventions should thus target explicit digital skills training, instead of an exclusively technical requirement, and focus on reform goals.

Training should cover the use of the system as well as the governance aspects of using digital tools, such as interpreting data, decision making and redesigning workflows. To enable effective engagement with digital systems, both clinicians and managers will need support to get involved in digital systems in a meaningful way, rather than simply as a compliance activity. This is especially significant in cluster-based systems where there is a strong need for coordination between services, which is dependent on shared information systems.

### 6.5 Balancing localization with knowledge retention for sustainability

All of these policy implications highlight the need to consider balance between localization goals and mechanisms that ensure institutional knowledge and professional expertise. The workforce sustainability of the SHSTP will rely on the ability to increase national participation in the system, as well as on the continuity, capability and confidence of the workforce in the system. Expatriate experts can be withdrawn prematurely if there are no appropriate support structures in place which can jeopardize reform progress, or they can be over-reliant on informal arrangements, leading to burnout and instability.

A sustainable approach to workforce governance thus needs to be planned: formalizing mentorship, building leaders, ensuring fair appraisal, and linking digital capacity-building to organizational needs. These policy actions can be used to reinforce implementation of the policies, while also staying true to the ultimate aim of Vision 2030 (Table 2).

**Table 2:** The recommendations matrix for workforce governance of the SHSTP.

Identified issue	Proposed policy action	Responsible actor	Expected outcome
Informal and uneven knowledge transfer	Establish structured mentorship programs pairing Saudi and expatriate clinicians/managers, with protected time and recognition	Ministry of Health; Health clusters	Improved knowledge transfer and workforce capability
Leadership role strain and variable preparedness	Develop an integrated leadership development pipeline aligned with cluster governance and reform priorities	Ministry of Health; Training bodies	Better-prepared leaders and clearer leadership roles
Perceived inequities in appraisal and progression	Implement transparent, standardized appraisal and career progression frameworks across clusters	Ministry of Health; Hospital management	Greater fairness and transparency in appraisal and progression
Administrative burden of digital systems	Provide targeted digital governance and data-use training aligned with reform objectives	Health clusters; Training bodies	More effective digital use and reduced administrative burden
Risk of knowledge loss during localization	Sequence localization targets with retention and transition mechanisms to preserve institutional expertise	Ministry of Health	Retention of institutional expertise during localization

### 6.6 Limitations

This study has several limitations. First, the qualitative sample comprised 53 participants, including 30 physicians and 23 patients, recruited from SHSTP-affected healthcare settings. Although purposive sampling was used to capture variation in professional roles, nationality and care settings, the sample was not intended to be statistically representative of the Saudi health system. The findings should therefore be interpreted as contextually grounded accounts of workforce governance rather than as evidence of system-wide experiences. Second, patient participants were included to provide a service-level perspective on the consequences of workforce governance; their accounts should not be interpreted as direct evidence of internal workforce dynamics. Third, patient recruitment involved hospital gatekeepers, which may have influenced access to eligible participants and introduced the possibility of selection bias. Finally, the analysis is situated within the Saudi health-sector reform context, and the transferability of the findings to other health systems will depend on similarities in workforce composition, governance arrangements, localization policies and organizational contexts.

## 7 Conclusion

The findings of this study indicate that the governance of the workforce is not an automatic consequence of policy design and/or organization restructuring as part of the Saudi Health Sector Transformation Program (SHSTP). Instead, reform is negotiated on a day-to-day basis, in clinical teams, managerial relationships, and in the work of adjusting to new systems of accountability. The results show that collaboration, authority relations, leadership practices, capacity development and motivation are all interrelated and influence the implementation of reform at the policy/service delivery interface.

The study foregrounds actors and process, providing a practice-oriented account of the implementation of reform in a hybrid workforce. Formal structures and digital performance systems can offer a framework for transformation, but the way that clinicians and managers behave, understand their roles, manage tensions and coordinate care will be key to achieving stable, credible organizational change that meets reform goals. The thematic network in this paper highlights the fact that these dynamics are not stand-alone problems but rather are a system of governance where changes in one aspect (e.g., leadership) affect others (e.g., collaboration, retention).

One of the main contributions of this paper is to highlight cultural and professional hybridity as a condition of governance and not a transitional anomaly. The findings suggest that expatriate expertise is still a valuable organizational resource in the context of reform, as is the case for localization agendas and leadership development in the long-term. Governance models that assume a linear pathway from expatriates to full localization may fail to account for the actual experiences of mixed governance teams, informal governance and knowledge transfer processes already in place that maintain service continuity. More powerful models acknowledge hybridity as an operating context and take a proactive approach to it, with a structured mentoring approach, clear authority structures and fair development-focused appraisal systems.

By combining technology and organizational culture with Walt and Gilson's triangle, the adapted policy analysis framework used in this study provides an explanation for the differences in experiences of reform in the various settings, and for the ways in which digital governance can be used to hold actors to account and yet at the same time create additional administrative pressure. From a practical perspective, this means that the sustainability of SHSTP reforms will rely on developing workforce governance mechanisms that enable capability, legitimacy and continuity within the system as well.

Finally, the effectiveness of the SHSTP as a transformative program will depend on how far governance arrangements recognize and enable the human work of reform: negotiating authority, sustaining collaboration and building capacity in ways that hold on to knowledge and drive localization.

## Data Availability

The aggregated and anonymized datasets supporting the findings of this study are provided as supplementary materials. Additional underlying data are available from the corresponding author on reasonable request, subject to institutional and ethical restrictions.
