# Correction: Negotiating reform from within: workforce governance and the expatriate–Saudi interface in the SHSTP era

**DOI:** 10.3389/fpubh.2026.2007011

**Published:** 2026-10-22

**Authors:** Ahmed Alshehri, Asaad Abdulrahman Abduljawad

**Affiliations:** 1 Healthcare Management and Medical Informatics Department, College of Health Sciences, Umm Al-Qura University, Makkah, Saudi Arabia; 2 Department of Public Health, College of Health Sciences, Umm Al-Qura University, Makkah, Saudi Arabia

**Keywords:** expatriate healthcare workforce, health system reform, leadership, qualitative research, Saudi Health Sector Transformation Program (SHSTP), Saudization, vision 2030, workforce governance

The order of authors in the author list and order of affiliations of the published paper was erroneously given as:

Asaad Abdulrahman Abduljawad^1^^*^ and Ahmed Alshehri^2^

^1^Department of Health Care Management and Health Informatics, College of Health Sciences, Umm Al-Qura University, Makkah, Saudi Arabia

^2^Department of Public Health, College of Health Sciences, Umm Al-Qura University, Makkah, Saudi Arabia

The correct author list and affiliation list reads:

Ahmed Alshehri^1^ and Asaad Abdulrahman Abduljawad^2^^*^

^1^Healthcare Management and Medical Informatics Department, College of Health Sciences, Umm Al-Qura University, Makkah, Saudi Arabia

^2^Department of Public Health, College of Health Sciences, Umm Al-Qura University, Makkah, Saudi Arabia

The original version of this article has been updated.

